# Dyskalemia, its patterns, and prognosis among patients with incident heart failure: A nationwide study of US veterans

**DOI:** 10.1371/journal.pone.0219899

**Published:** 2019-08-08

**Authors:** Kunihiro Matsushita, Yingying Sang, Chao Yang, Shoshana H. Ballew, Morgan E. Grams, Josef Coresh, Miklos Z. Molnar

**Affiliations:** 1 Johns Hopkins University, Baltimore, MD, United States of America; 2 University of Tennessee Health Science Center, Memphis, TN, United States of America; Kurume University School of Medicine, JAPAN

## Abstract

**Background:**

Although hypokalemia has been viewed as a significant concern among patients with heart failure (HF), recent advances in HF management tend to increase the risk of hyperkalemia.

**Objective:**

To characterize contemporary data regarding correlates and prognostic values of dyskalemia in patients with HF.

**Design, setting, and participants:**

In cross-sectional and longitudinal analyses, we studied 142,087 patients with newly diagnosed HF in US nationwide Veterans Administration database from 2005 through 2013.

**Exposures:**

Demographic characteristics, laboratory variables, comorbidities, and medication use for the analysis of correlates of dyskalemia as well as potassium level in the analysis of mortality.

**Main Outcomes and Measures:**

Dyskalemia and mortality.

**Results:**

Hypokalemia (<3.5 mmol/L) at baseline was observed in 3.0% of the population, whereas hyperkalemia (≥5.5 mmol/L) was seen in 0.9%. An additional 20.4% and 5.7% had mild hypokalemia (3.5–3.9 mmol/L) and mild hyperkalemia (5.0–5.4 mmol/L). Key correlates were black race, higher blood pressure, and use of potassium-wasting diuretics for hypokalemia, and lower kidney function for hyperkalemia. Baseline potassium levels showed a U-shaped association with mortality, with the lowest risk between 4.0–4.5 mmol/L. With respect to potassium levels over a year after HF diagnosis, persistent (>50% of measurements), intermittent (>1 occurrence but ≤50%), and transient (1 occurrence) hypo- and hyperkalemia were also related to increased mortality in a graded fashion regardless of the aforementioned thresholds for dyskalemia. These dyskalemic patterns were also related to other clinical actions and demands such as emergency room visit.

**Conclusions:**

Potassium levels below 4 mmol/L and above 5 mmol/L at and after HF diagnosis were associated with poor prognosis and the clinical actions. HF patients (particularly with risk factors for dyskalemia like black race and kidney dysfunction) may require special attention for both hypo- and hyperkalemia.

## Introduction

Potassium is an important element in cell metabolism and membrane excitability, and thus its maintenance within a therapeutic range is essential for a regular heart rhythm.[1] Dyskalemia (both hypokalemia and hyperkalemia) is particularly relevant to patients with heart failure (HF), given associated neurohormonal activation and the use of HF drugs (e.g., renin angiotensin system inhibitors and diuretics) which can affect potassium homeostasis and increase risks of life-threatening arrhythmias.[1, 2] Historically, hypokalemia, compared to hyperkalemia, has been considered more concerning among patients with HF due to the associated risk of ventricular fibrillation and digoxin toxicity.[3–5] However, recent studies support the use of renin-angiotensin aldosterone inhibitors as a cornerstone of HF management, and these medications increase the risk of hyperkalemia. Therefore, contemporary data regarding the prognostic values of dyskalemia in HF patients are needed.

In this context, it is of note that most recent relevant studies are relatively small,[3] mainly focus on dyskalemia related to specific HF drugs,[6] only explore HF after myocardial infarction,[7] or use data from clinical trials with selected populations,[8–10] and thus have limited generalizability. A recent Israeli study overcame these caveats by analyzing data from a large health management organization.[11] However, this study included heterogeneous patients with both incident and chronic HF and thus is susceptible to survival bias for chronic cases. This aspect would be particularly important for a clinical condition with poor prognosis such as HF.[12] Also, to our knowledge, data regarding the patterns of potassium levels over time (e.g., transient, intermittent, or persistent[13]) and actual clinical actions after dyskalemia in HF patients are lacking. Therefore, to comprehensively assess the prognostic impact of dyskalemia and its patterns in real-world clinical settings, we investigated patients with newly diagnosed HF in a nationwide study of US veterans.

## Methods

### Study population

Our study used data from a retrospective cohort study aiming to examine risk factors for incident chronic kidney disease (CKD) in US veterans with preserved kidney function at baseline (the Racial and Cardiovascular Risk Anomalies in CKD [RCAV] study).[14] The detailed algorithm for constructing the RCAV cohort has been described previously.[14, 15] Briefly, using the national Veterans Affairs (VA) Corporate Data Warehouse LabChem data files to extract serum creatinine measured between October 1, 2004 and September 30, 2006, 3,582,478 veterans with baseline estimated glomerular filtration rate (eGFR) ≥60 mL/min/1.73m^2^ were identified.[16] eGFR was calculated using the Chronic Kidney Disease Epidemiology Collaboration (CKD-EPI) creatinine equation.[17] After excluding veterans with missing ICD (International Classification of Diseases) 9 codes to define comorbidities or outcomes (n = 11,311) or with erroneous data (n = 66,435), 3,504,732 patients were included in RCAV.

For the current study, from those 3,504,732 US veterans in RCAV, we identified 224,858 patients with a hospitalization or two outpatient encounters with ICD-9 code 428 for HF during follow-up through August 2013 (S1 Fig).[18–20] Of these, 46,206 cases were considered prevalent cases (HF diagnosis in the first year from cohort entry) and were excluded.[21] Of the remaining 178,652 patients, we further excluded 36,565 veterans without any outpatient potassium measurements six months prior to HF diagnosis, information on covariates, or linkage to all-cause mortality, leaving 142,087 patients with newly diagnosed HF for this study.

### Baseline variables at HF diagnosis

In RCAV, information on demographic characteristics, laboratory variables, comorbidities, and medication use was obtained from various national VA research data files as previously described.[14, 15, 22] Laboratory data including serum potassium were based on the Decision Support System National Data Extracts Laboratory Results file.[22] Diabetes mellitus, coronary artery disease, stroke, peripheral artery disease, and atrial fibrillation were identified as comorbidities based on ICD codes and procedure codes, as appropriate. Information on the following medications was captured within six months prior to incident HF: angiotensin converting enzyme inhibitors or angiotensin receptor blockers, loop or thiazide (potassium-wasting) diuretics, potassium-sparing diuretics, beta-blockers, other anti-hypertensive medications, insulin, other anti-diabetic medications, statins, digitalis, and other anti-arrhythmic medications. We allowed a period of 12 months prior to HF to capture baseline information on eGFR based on the CKD-EPI creatinine equation, blood pressure, and body mass index at outpatient settings.

### Levels of potassium at HF diagnosis and their pattern after HF diagnosis

In the primary analysis, baseline potassium levels were defined as the average value in the outpatient setting within six months prior to HF diagnosis. We also conducted a sensitivity analysis in which baseline potassium was defined as the average within three months prior to HF diagnosis. Hypokalemia and hyperkalemia were defined as <3.5 and ≥5.5 mmol/L,[6, 23] respectively. We also explored mild hypo- and hyperkalemia, defined as <4 and ≥5 mmol/L,[13] respectively. Subsequently, we examined the pattern of dyskalemia over 1- and 2-year periods after HF diagnosis. As done previously,[13] patterns of hyperkalemia and hypokalemia were defined as never, transient (only 1 occurrence), intermittent (>1 occurrence but ≤50% of the potassium measurements), and persistent (>50% of potassium measurements).

### Follow-up and mortality

Information on mortality was based on the Vital Status Files (a registry containing dates of death and any encounters from all available sources in the VA system).[22] Patients were followed until death, the date of the last health care or administrative VA encounter, as documented in the Vital Status Files, or the end of follow-up (August 14, 2013).

### Actions taken after dyskalemia

We examined the following actions taken after hyperkalemia and hypokalemia[13]: emergency room visit within 7 days, repeat potassium measurement within 14 days, and initiation of kayexalate, initiation or discontinuation of potassium-wasting diuretics, initiation or discontinuation of potassium-sparing diuretics, initiation or discontinuation of renin-angiotensin system inhibitors, and initiation or discontinuation of oral potassium chloride within 60 days.[13] We considered that these drugs were discontinued when the last prescription ended within 60 days after the identification of dyskalemia and there was no follow-up prescription in 90 days after the end date of the last prescription.[13] These actions were evaluated for the respective two cutpoints for hyperkalemia and hypokalemia after initial HF diagnosis.

### Statistical analysis

Baseline characteristics of patients with incident HF were summarized according to baseline potassium levels of <3.5, 3.5–3.9, 4.0–4.9, 5.0–5.4, and ≥5.5 mmol/L. Multinomial logistic regression models were used to identify correlates of baseline hypokalemia and hyperkalemia. We estimated survival after incident HF across baseline potassium categories using the Kaplan-Meier method. Using Cox’s proportional hazards regression models, we evaluated whether the associations of baseline potassium levels are independent of potential confounders. We ran two models: Model 1, unadjusted and Model 2, adjusted for age, gender, race, blood pressure, body mass index, diabetes, and a history of coronary heart disease, stroke, peripheral artery disease, and atrial fibrillation, and use of angiotensin-converting enzyme inhibitor/angiotensin receptor blockers, potassium-wasting diuretics, potassium-sparing diuretics, beta-blockers, use of other anti-hypertensive medications, insulin, other anti-diabetic medications, statins, digoxin, and anti-arrhythmic medications at baseline.

Subsequently, we identified patterns (never, transient, intermittent, and persistent) of dyskalemia over 1- and 2-year period after HF diagnosis and evaluated whether these patterns were associated with mortality independently of potential confounders using Cox models. Since frequency of serum potassium measurements might affect the detection of potassium patterns, we included measurement frequency (<2, 2 to <4 and ≥4 measurements per year) in both Models 1 and 2 for this analysis. We also quantified the clinical actions taken after first dyskalemia seen after incident HF and contrasted with those in a control group. The control group was matched on the frequency of potassium measurement (the same categories shown above), since the frequency of serum potassium measurements might affect the detection of dyskalemia and be related to patterns of subsequent clinical actions. All analyses were performed using Stata 14.

## Results

### Baseline characteristics among incident HF patients

Of 142,087 patients with incident HF, 3.0% (n = 4,320) had baseline serum potassium levels <3.5 mmol/L and 0.9% (n = 1,303) had ≥5.5 mmol/L (the primary definition of hypokalemia and hyperkalemia, respectively) (Table 1). Additional 20.4% (n = 29,032) and 5.7% (n = 8,124) had mild hypokalemia of 3.5–3.9 mmol/L and mild hyperkalemia of 5.0–5.4 mmol/L, respectively. Male sex, non-black race, lower kidney function, diabetes, a history of peripheral artery disease, use of renin angiotensin system inhibitors, insulin, and oral anti-diabetic drugs, and no use of other anti-hypertensive drugs and potassium-wasting diuretics were generally associated with higher potassium levels. An inverse U- or J-shaped association was seen across potassium categories for a history of coronary disease, stroke, atrial fibrillation, and use of beta blockers, statins, digoxin, and other anti-arrhythmic drugs, with the highest prevalence of each condition in the potassium category of 4.0–4.9 mmol/L or 5.0–5.4 mmol/L. The prevalence of use of potassium-sparing diuretics demonstrated a U-shaped pattern with the lowest prevalence in the potassium category of 4.0–4.9 mmol/L and higher prevalence in both hypo- and hyperkalemia categories.

**Table 1:** Baseline characteristics according to baseline potassium levels (N = 142,087).

	Potassium levels (mmol/L)				
Characteristic	<3.5	3.5–3.9	4.0–4.9	5.0–5.4	≥5.5
N (%)	4,320 (3.0%)	29,032 (20.4%)	99,308 (69.9%)	8,124 (5.7%)	1,303 (0.9%)
Age (years)	66.9±11.0	68.7±11.1	70.2±10.7	70.6±10.3	69.5±10.4
Female	3.8%	3.1%	1.9%	1.4%	1.6%
Black race	31.8%	23.4%	13.7%	9.9%	11.8%
eGFR (mL/min/1.73 m^2^)	73.2±22.0	73.9±20.2	70.3±19.6	61.3±21.0	53.9±23.3
Systolic blood pressure (mmHg)	135.8±23.6	132.9±21.1	129.8±19.9	129.4±20.4	129.5±22.7
Body mass index (kg/m^2^)	31.1±7.9	31.0±7.6	30.7±7.4	30.2±7.3	29.8±7.6
Diabetes	49.4%	49.1%	53.0%	61.4%	66.4%
Hypertension	93.8%	92.5%	90.6%	91.6%	91.4%
History of coronary artery disease	55.5%	60.1%	67.0%	68.9%	65.5%
History of cerebrovascular disease	22.0%	22.5%	23.2%	23.4%	22.1%
History of peripheral artery disease	19.3%	20.6%	23.8%	27.5%	26.9%
History of atrial fibrillation	27.7%	29.8%	30.5%	28.6%	23.5%
Use of ACEI/ARB	56.9%	59.9%	65.1%	68.8%	68.0%
Use of loop/thiazide diuretics	68.2%	63.1%	54.5%	51.8%	50.1%
Use of K-sparing diuretics	13.8%	10.6%	9.1%	10.9%	14.0%
Use of beta-blockers	59.5%	61.2%	64.2%	66.5%	64.4%
Use of other anti-hypertensive medications	61.9%	56.8%	48.0%	45.5%	46.5%
Use of insulin	21.0%	20.9%	23.5%	29.6%	32.4%
Use of oral anti-diabetic medications	28.7%	29.5%	34.0%	39.8%	40.1%
Use of statins	40.9%	43.3%	45.6%	46.4%	42.4%
Use of anti-arrhythmic medications	3.3%	3.8%	4.3%	4.2%	3.4%
Use of digoxin	8.4%	9.7%	11.8%	12.5%	11.8%

Numbers were shown as mean±SD for continuous variables or % for dichotomous variables. ACEI = angiotensin-converting enzyme inhibitor, ARB = angiotensin receptor blockers, K = potassium.

In multivariable multinomial logistic regression analysis, many of these factors were significantly associated with hypo- and hyperkalemia (S1 Table). With <3.5 mmol/L as a threshold for hypokalemia, the following factors demonstrated strong significant associations, with Z-score <-4.06 or >4.06 (p <5*10^−5^): younger age, female sex, black race, higher systolic blood pressure, no history of coronary disease, no use of angiotensin-converting enzyme inhibitor/angiotensin receptor blockers, use of potassium-wasting diuretics, use of potassium-sparing diuretics, no use of beta blockers, use of other anti-hypertensive drugs, no use of insulin, and no use of digoxin. Of these, younger age, black race, higher systolic blood pressure, no use of angiotensin-converting enzyme inhibitor/angiotensin receptor blockers, use of potassium-wasting diuretics, and use of other anti-hypertensive drugs were particularly strongly associated with hypokalemia with Z-score <-10 or >10. With ≥5.5 mmol/L as a threshold for hyperkalemia and the threshold of Z-score <-4.06 or >4.06, the following factors showed strong positive associations: younger age, non-black race, lower eGFR, lower body mass index, diabetes, no use of potassium-wasting diuretics, use of potassium-sparing diuretics, and no use of other anti-hypertensive drugs. Of these, only reduced eGFR below 60 ml/min/1.73m^2^ was associated with a Z-score >10. Similar results were observed when <4 and ≥5 mmol/L were used as a threshold for hypo- and hyperkalemia, respectively (S2 Table).

### Baseline potassium levels at incident HF and mortality risk

**Fig 1:**
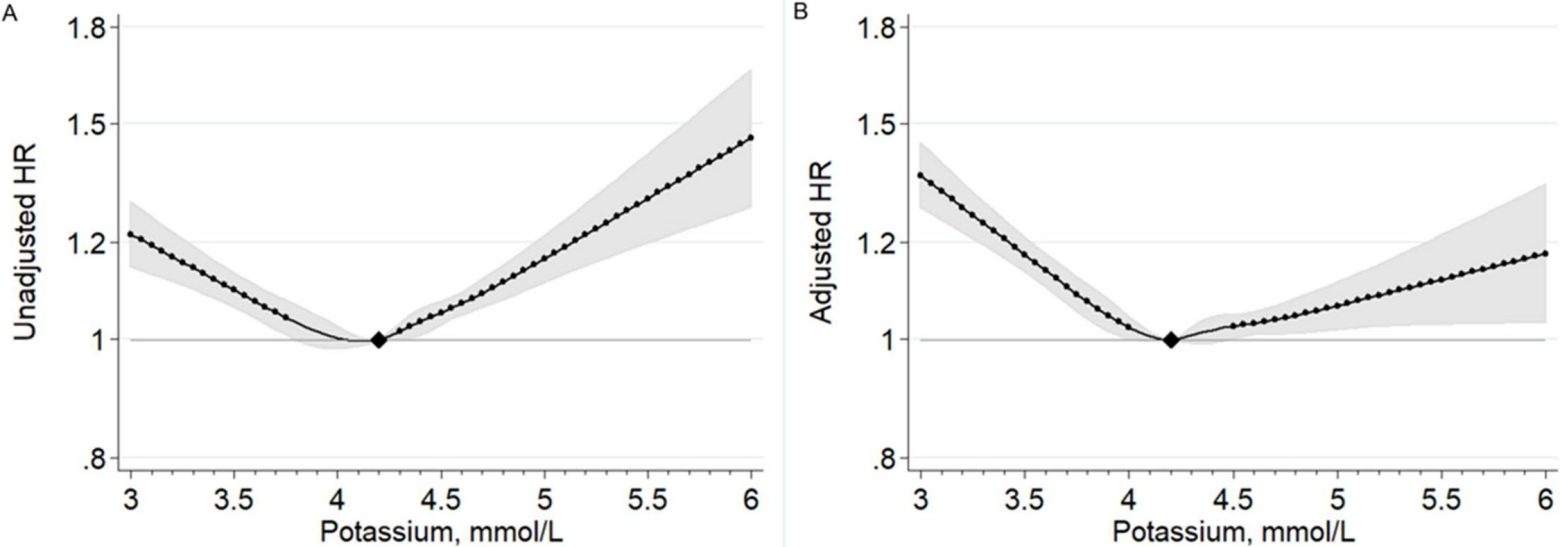
Unadjusted (A) and adjusted* (B) hazard ratio of mortality after incident heart failure according to baseline serum potassium levels in the range of 0.02 to 99.8 percentiles. Diamond indicates the reference point at 4.2 mmol/L; dot, statistical significance compared to the reference point; shade, 95% confidence intervals. Knots were put at 3.6, 4.0, 4.2, 4.4, 4.6, and 5.0 mmol/L. *Adjusted for age, gender, race, blood pressure, body mass index, diabetes, a history of coronary heart disease, stroke, peripheral artery disease, and atrial fibrillation, and use of angiotensin-converting enzyme inhibitor/angiotensin receptor blockers, potassium-wasting diuretics (loop and thiazide), potassium-sparing diuretics, beta-blockers, use of other anti-hypertensive medications, insulin, other anti-diabetic medications, statins, digoxin, and anti-arrhythmic medication.

During a median follow-up of 3.1 years after incident HF, 53,947 deaths occurred. In our study population with incident HF, the five year survival estimate was 56.2% (S2 Fig). In an unadjusted model, the lowest mortality risk was observed at a serum potassium level of 4.0–4.2 mmol/L (Fig 1A). The mortality risk steadily increased below and above this level (dots in Fig 1 represent significantly elevated risk). In this unadjusted model, the mortality risk at potassium level of 3.0 mmol/L was equivalent to that at potassium level of ~5.2 mmol/L, and 3.5 mmol/L to ~4.8 mmol/L. From another perspective, the mortality risk at potassium level of 5.5 mmol/L was equivalent to that at 2.7 mmol/L. The shape of associations remained similar after the adjustment for potential confounders, although the risk gradient became shallower at higher potassium levels but steeper at lower potassium levels (Fig 1B). The lowest mortality risk was seen around potassium levels of 4.2 mmol/L. In this fully adjusted model, potassium levels at 3.5 mmol/L showed a similar hazard ratio as potassium levels around 6 mmol/L. The associations were similar when baseline potassium levels were based on data within three months prior to incident HF (data not shown). Also, the shape of associations were generally consistent across kidney function (except eGFR <30 ml/min/1.73m^2^) (S3 Fig) and regardless of the use of potassium-sparing diuretics (S4 Fig) or potassium-wasting diuretics (S5 Fig).

### Patterns of dyskalemia after incident HF and clinical consequences

Of 142,087 patients with incident HF, 118,477 patients had at least a measurement of serum potassium levels within one year after incident HF. Of these, 6.5% (n = 7,740) had transient hypokalemia (<3.5 mmol/L), 2.7% (n = 3,207) intermittent hypokalemia, and 1.8% (n = 2,456) persistent hypokalemia within the first year of incident HF. In the same period after incident HF, transient, intermittent, and persistent hyperkalemia (≥5.5 mmol/L) were seen in 3.3% (n = 3,886), 1.1% (n = 1,316), and 0.4% (n = 486).

Both persistent hypokalemia (<3.5 mmol/L) and hyperkalemia (≥5.5 mmol/L) over one year after incident HF were associated with increased mortality, with slightly stronger associations for a given pattern of hyperkalemia compared to the counterpart of hypokalemia when only adjusted for the frequency of potassium measurements (Model 1 in Table 2). Once we accounted for other potential confounders (Model 2), both hypo- and hyperkalemia patterns were similarly associated with higher risk of mortality, with the highest risk in the persistent pattern (adjusted HR 1.6–1.7) followed by intermittent and transient patterns (adjusted HR 1.3–1.5). Similar, but slightly weaker, associations were observed when <4 and ≥5 mmol/L were used as thresholds (S3 Table) or hypokalemia (<3.5 mmol/L) and hyperkalemia (≥5.5 mmol/L) patterns over two years after incident HF were assessed, except slightly stronger associations for their persistent pattern (S4 Table).

**Table 2:** Adjusted hazard ratio (95% confidence interval) of mortality for transient, intermittent, and persistent hypo- and hyperkalemia over one year after incident HF (vs. no hypo- or hyperkalemia as referent) (N = 118,477).

Model	K level (mmol/L)	Dyskalemia pattern			
		0 (no)	1 (transient)	2 (intermittent)	3 (persistent)
1	K<3.5	Referent	1.24 (1.19,1.29)	1.34 (1.27,1.42)	1.32 (1.23,1.41)
2	K<3.5	Referent	1.30 (1.25,1.35)	1.49 (1.41,1.58)	1.56 (1.46,1.67)
1	K≥5.5	Referent	1.46 (1.39,1.54)	1.47 (1.35,1.59)	2.00 (1.77,2.25)
2	K≥5.5	Referent	1.34 (1.28,1.41)	1.30 (1.19,1.41)	1.66 (1.48,1.88)

Model 1 adjusted for frequency of potassium measurement. Model 2 additionally adjusted for age, gender, race, estimated glomerular filtration rate, systolic blood pressure, body mass index, diabetes, history of coronary artery disease, history of cerebrovascular disease, history of peripheral artery disease, and history of atrial fibrillation, use of ACEI/ARB, use of loop/thiazide diuretics, use of K-sparing diuretics, use of beta-blockers, use of other anti-hypertensive medications, use of insulin, use of other anti-diabetic medications, use of statins, use of anti-arrhythmic drugs, and use of digoxin. ACEI = angiotensin-converting enzyme inhibitor, ARB = angiotensin receptor blockers, K = potassium.

Approximately 14% of participants with a measurement of potassium levels <3.5 or ≥5.5 mmol/L within a year after HF diagnosis visited an emergency room within a week after detection of dyskalemia (Table 3). Among HF patients experiencing hyperkalemia ≥5.5 mmol/L, 43.4% received repeated measurement of serum potassium levels within two weeks, whereas 29.4% of hypokalemic patients <3.5 mmol/L received repeated potassium exam. Discontinuation of angiotensin-converting enzyme inhibitor/angiotensin receptor blockers, potassium-wasting diuretics, or potassium-sparing diuretics was more commonly seen in those with hypokalemia and hyperkalemia compared to their respective referent control. Initiation of potassium-sparing diuretics was most often seen in those with hypokalemia although the proportion was low (5.2%). Discontinuation of beta-blockers was more frequently seen in hypokalemia than in control whereas their initiation was more often observed in hyperkalemia. Kayexalate was prescribed in 19.0% of hyperkalemia ≥5.5 mmol/L, whereas potassium chloride was prescribed in 15.7% with hypokalemia <3.5 mmol/L. Generally, results were similar when the potassium pattern over 2 years after incident HF was investigated (S5 Table).

**Table 3:** Actions taken after experiencing hypo- or hyperkalemia over one year after incident heart failure.

	Hypokalemia (<3.5 mmol/L)		Hyperkalemia (≥5.5 mmol/L)	
Action taken	Case	Control	Case	Control
Emergency room visit within 7 days	14.2% (792/5585) ^‡^	4.9% (2937/60378)	13.9% (592/4247) ^‡^	6.1% (5864/95612)
Repeated potassium measurement within 14 days	29.4% (1641/5585) ^‡^	7.3% (4403/60378)	43.4% (1844/4247) ^‡^	8.8% (8377/95612)
Discontinuation of ACEI/ARB within 60 days	14.1% (399/2822) ^‡^	8.2% (2829/34359)	14.2% (395/2791) ^‡^	8.2% (4299/52415)
Initiation of ACEI/ARB within 60 days	10.7% (297/2763)	10.2% (2662/26019)	10.3% (150/1456)	10.1% (4378/43197)
Discontinuation of diuretics within 60 days	15.8% (570/3601) ^‡^	12.0% (3296/27565)	16.5% (404/2448) ^‡^	12.2% (5915/48324)
Initiation of diuretics within 60 days	16.2% (321/1984) ^‡^	10.1% (3327/32813)	14.8% (267/1799) ^‡^	10.8% (5097/47288)
Discontinuation of K-sparing diuretics within 60 days	21.7% (117/540) ^‡^	12.6% (694/5527)	26.7% (219/820) ^‡^	12.9% (1113/8611)
Initiation of K-sparing diuretics within 60 days	5.2% (261/5045) ^‡^	1.8% (980/54851)	2.6% (90/3427)	2.2% (1950/87001)
Discontinuation of beta-blockers within 60 days	9.3% (310/3337) ^‡^	6.7% (2303/34509)	7.4% (206/2783)	7.0% (3781/53670)
Initiation of beta-blockers within 60 days	10.9% (246/2248)	10.0% (2589/25869)	12.2% (178/1464) ^†^	10.0% (4185/41942)
Initiation of kayexalate within 60 days	0.7% (5/728) ^†^	2.7% (183/6731)	19.0% (257/1355) ^‡^	0.1% (9/6839)
Initiation of K supplement within 60 days	15.7% (452/2880) ^‡^	4.2% (776/18608)	3.7% (59/1611)	4.5% (1692/37502)

ACEI = angiotensin-converting enzyme inhibitor, ARB = angiotensin receptor blockers, K = potassium. Control was matched on frequency of potassium measurements (<2, 2 to <4 and ≥4 measurements per year).

^†^p<0.01,

^‡^p<0.001

## Discussion

This nationwide cohort of US veterans demonstrated that ~4% of patients with incident HF had dyskalemia (<3.5 or ≥5.5 mmol/L) at the time of diagnosis. The prevalence went up to 30% if we used the threshold of <4.0 or ≥5.0 mmol/L. Although we observed several correlates for baseline dyskalemia in this population, younger age, black race, higher systolic blood pressure, and use of potassium-wasting diuretics were particularly strongly associated with hypokalemia and lower eGFR with hyperkalemia. Both baseline hypokalemia and hyperkalemia at incident HF were significantly associated with increased mortality in this clinical population, with the lowest risk at potassium 4–4.5 mmol/L. Moreover, dyskalemia patterns after incident HF were also associated with mortality, with the highest risk in persistent (>50% of potassium measurements) hypokalemia and hyperkalemia. Both hypokalemia and hyperkalemia resulted in clinical actions or demands such as an emergency room visit, discontinuation/initiation of specific evidence-based drugs for HF management, and the use of potassium chloride or kayexalate, respectively.

Although several studies have reported the associations of both hypokalemia and hyperkalemia with poor prognosis in patients with HF, our study has a few unique aspects. First, to our knowledge, this is one of the largest studies exploring the prognostic value of potassium in the context of incident HF. Second, this study comprehensively examined the prognostic value of baseline potassium at HF diagnosis and potassium patterns after the diagnosis. The evaluation of potassium patterns and prognosis is novel in this clinical population. Third, our study uniquely captured clinical actions taken after dyskalemia over up to two years after the initial diagnosis of HF. Finally, our study extensively adjusted for comorbidities and medications.

An increased risk of ventricular fibrillation due to hypokalemia has been established.[3, 4] Hypokalemia is also concerning for increasing the risk of digoxin toxicity. Hence, empirically, cardiologists have been more concerned about hypokalemia than hyperkalemia. Indeed, some investigators recommend that serum potassium level should be maintained slightly higher than usual at the range of 4.5–5.5 mmol/l in patients with HF.[24] In this context, it is of importance that our study demonstrated the lowest mortality risk at baseline potassium levels of 4–4.5 mmol/L, as well as significantly elevated mortality risk related to potassium ≥5 mmol/L, even when only transient after the HF diagnosis (S3 Table). Importantly, this finding is in line with other recent reports showing the best prognosis among patients with HF at potassium levels 4–4.9 mmol/L.[6, 7, 10] Nonetheless, our results contrasted different levels of serum potassium for mortality risk and thus should not be interpreted as a potential harm of specific HF drugs altering potassium levels (e.g., our study was not designed to compare potassium-sparing diuretics vs. placebo or standard care).

In addition to mortality, dyskalemia events seemed to result in clinical actions. Specifically, both hypo- and hyperkalemia increased the visits to emergency rooms and repeated blood tests shortly after its recognition. Hypokalemia triggered the prescription of potassium-sparing diuretics and potassium supplements, whereas hyperkalemia resulted in the discontinuation of potassium-sparing diuretics and initiation of kayexalate, as anticipated. Actions related to other evidence-based medications, angiotensinogen converting enzyme inhibitors/angiotensin receptor blockers and potassium-sparing diuretics were different between dyskalemia and control but not that different between hypokalemia and hyperkalemia. Although this is somewhat counterintuitive, this may reflect the unstable conditions behind dyskalemia, rather than potassium level per se, influencing clinical actions. Thus, randomized controlled design would be ideal to evaluate whether modifications of these drugs in dyskalemia influence prognosis in HF patients.

Although more than the control groups, some of those actions might appear to be infrequently taken despite dyskalemia. However, we should keep in mind that there are not much previous data for effective comparison. Nonetheless, when we contrasted clinical actions after hyperkalemia with a previous study focusing on hypertensive patients (not restricted to HF patients) in a regional healthcare management organization,[13] the frequency of emergency visits and kalyexalate prescription was much higher in our study population (13.9% vs. 3.1% and 19.0% vs. 4.7%, respectively). The frequency of repeat potassium measurement was similar in the two studies. In contrast, the discontinuation of angiotensinogen converting enzyme inhibitors/angiotensin receptor blockers and potassium-sparing diuretics was much less in our study population (14.2% vs. 24.3% and 26.7% vs. 48.5%). This may reflect physicians’ desire to continue these evidence-based medications in HF patients. Nevertheless, we would need more data to obtain conclusive interpretations.

Black race and the use of potassium-wasting diuretics were particularly strongly related to hypokalemia, which is consistent with previous reports.[25, 26] The strong association of potassium-wasting diuretics and hypokalemia is reasonable. For the association between black race and hypokalemia, we recently reported a potential genetic aspect behind this racial difference.[25] The most potent correlate of hyperkalemia (in terms of Z-score) was reduced kidney function in our study. Some other comorbidities were also related to dyskalemia (e.g., diabetes with hyperkalemia), and thus HF patients with demographic or clinical conditions noted above may require close monitoring of blood potassium levels.

There were several limitations in our study. First, the identification of HF cases was based on ICD code 428. Although this code is most commonly used for patients with HF diagnosis[27] and has shown decent positive predictive value of 77%, some misclassification is likely. Second, we did not have data on ejection fraction and thus could not address whether results are different between HF with preserved vs. reduced ejection fraction. Third, reflecting the nature of the data source, most patients were men. So, confirmatory studies in women would be warranted. Fourth, the measurement of potassium levels was based on routine care and thus susceptible to indication bias, although we tried to account for the frequency of measurement when we assessed potassium patterns after HF diagnosis. Fifth, although a number of patients had reduced kidney function at HF diagnosis, our source data were originally selected for preserved kidney function. Thus, the prevalence of hyperkalemia is likely to be underestimated. Sixth, we relied on data that were not prospectively collected for this specific study. However, data collection was done for clinical purpose without knowledge for future outcomes. Moreover, the temporality of potassium levels and mortality is still prospective (so-called retrospective cohort study). Finally, as in any observation study, residual confounding is possible.

In conclusion, this nationwide administrative data study of US veterans demonstrates that dyskalemia is common in the initial course of HF, particularly when including mild cases. Both hypo- and hyperkalemia were associated with elevated mortality and the lowest risk was seen at potassium levels 4–5 mmol/L at and after incident HF. In addition, both hypo- and hyperkalemia resulted in clinical burden such as emergency room visits. Our results further support the importance of monitoring and managing potassium levels in patients with new diagnosis of HF, particularly among those of black race, taking potassium-wasting diuretics, and with reduced kidney function.

## Supporting information

S1 Table Relative risk ratio (95% CI) of hypo- (<3.5 mmol/L) and hyperkalemia (≥5.5 mmol/L) for potential correlates (N = 142,087). (DOCX)

S2 Table Relative risk ratio (95% confidence interval) of mild hypo- (<4.0 mmol/L) and hyperkalemia (≥5.0 mmol/L) for potential correlates (N = 142,087). (DOCX)

S3 Table Hazard ratio (95% confidence interval) of mortality for transient, intermittent, and persistent mild hypo- and hyperkalemia over one year after incident heart failure (no hypo- or hyperkalemia as referent) (N = 118,477). (DOCX)

S4 Table Hazard ratio (95% confidence interval) of mortality for transient, intermittent, and persistent hypo- and hyperkalemia over two years after incident heart failure (no hypo- or hyperkalemia as referent) (N = 121,542). (DOCX)

S5 Table Actions taken after experiencing hypo- and hyperkalemia over two years after incident heart failure. (DOCX)

S1 Fig Study flow diagram. (DOCX)

S2 Fig Kaplan-Meier survival estimates after incident HF. (DOCX)

S3 Fig Unadjusted (left) and adjusted* (right) hazard ratio of mortality after incident heart failure according to baseline serum potassium levels in the range of 0.02 to 99.8 percentiles by kidney function (≥90, 60–89, 30–59, and <30 ml/min/1.73m2). (DOCX)

S4 Fig Unadjusted (left) and adjusted* (right) hazard ratio of mortality after incident heart failure according to baseline serum potassium levels in the range of 0.02 to 99.8 percentiles by the use of potassium-sparing diuretics. (DOCX)

S5 Fig Unadjusted (left) and adjusted* (right) hazard ratio of mortality after incident heart failure according to baseline serum potassium levels in the range of 0.02 to 99.8 percentiles by the use of potassium-wasting diuretics. (DOCX)
