# Supplementary material for: Dyskalemia, its patterns, and prognosis among patients with incident heart failure: A nationwide study of US veterans
Source: PLoS One. 2019 Aug 8;14(8):e0219899. doi: 10.1371/journal.pone.0219899 (PMC6687136; doi:10.1371/journal.pone.0219899)
Supplement: S1 Table — (DOCX) [file pone.0219899.s001.docx]

S1 Table. Relative risk ratio (95% CI) of hypo- (<3.5 mmol/L) and hyperkalemia (≥5.5 mmol/L) for potential correlates (N=142,087)

| **Potential correlates** | **K<3.5** | **Z** | **K≥5.5** | **Z** |
| --- | --- | --- | --- | --- |
| Age (per 10 year increment) | 0.80 (0.78,0.83) | -13.08 | 0.85 (0.80,0.90) | -5.36 |
| Female | 1.42 (1.20,1.67) | 4.17 | 0.74 (0.48,1.15) | -1.34 |
| Black race | 1.93 (1.80,2.08) | 18.27 | 0.58 (0.49,0.70) | -5.88 |
| eGFR<60 (per 15 mL/min/1.73 m^2^ decrement) | 0.95 (0.90,1.01) | -1.61 | 2.18 (2.04,2.32) | 23.08 |
| eGFR≥60 (per 15 mL/min/1.73 m^2^ decrement) | 0.95 (0.92,0.98) | -3.11 | 1.49 (1.37,1.62) | 9.04 |
| Systolic blood pressure (per 20 mmHg increment) | 1.21 (1.18,1.25) | 13.08 | 0.94 (0.90,0.99) | -2.19 |
| Body mass index (per 5 kg/m^2^ increment) | 0.96 (0.94,0.98) | -3.59 | 0.88 (0.84,0.92) | -5.77 |
| Diabetes | 1.00 (0.92,1.10) | 0.11 | 1.45 (1.23,1.70) | 4.47 |
| History of coronary artery disease | 0.87 (0.81,0.93) | -4.14 | 0.92 (0.82,1.04) | -1.29 |
| History of cerebrovascular disease | 1.05 (0.97,1.13) | 1.28 | 0.86 (0.75,0.99) | -2.09 |
| History of peripheral artery disease | 0.86 (0.80,0.93) | -3.64 | 1.05 (0.92,1.20) | 0.75 |
| History of atrial fibrillation | 1.14 (1.06,1.23) | 3.48 | 0.75 (0.65,0.86) | -4.03 |
| Use of ACEI/ARB | 0.67 (0.63,0.72) | -11.64 | 1.26 (1.11,1.43) | 3.56 |
| Use of loop/thiazide diuretics | 1.87 (1.74,2.00) | 17.62 | 0.63 (0.56,0.71) | -7.56 |
| Use of K-sparing diuretics | 1.50 (1.37,1.65) | 8.79 | 1.46 (1.24,1.72) | 4.55 |
| Use of beta-blockers | 0.86 (0.80,0.92) | -4.51 | 0.93 (0.82,1.06) | -1.09 |
| Use of other anti-hypertensive medications | 1.56 (1.46,1.66) | 13.29 | 0.76 (0.68,0.86) | -4.54 |
| Use of insulin | 0.82 (0.75,0.90) | -4.35 | 1.09 (0.95,1.25) | 1.17 |
| Use of other anti-diabetic medications | 0.84 (0.77,0.92) | -3.80 | 1.22 (1.06,1.40) | 2.71 |
| Use of statins | 0.94 (0.88,1.01) | -1.80 | 0.83 (0.74,0.93) | -3.18 |
| Use of anti-arrhythmic drugs | 0.91 (0.77,1.09) | -1.02 | 0.78 (0.57,1.06) | -1.58 |
| Use of digoxin | 0.76 (0.68,0.85) | -4.65 | 1.19 (0.99,1.42) | 1.87 |
|  |  |  |  |  |
| eGFR=estimated glomerular filtration rate, ACEI=angiotensin-converting enzyme inhibitor, ARB=angiotensin receptor blockers, K=potassium. | | | | |
