# Supplementary material for: Dyskalemia, its patterns, and prognosis among patients with incident heart failure: A nationwide study of US veterans
Source: PLoS One. 2019 Aug 8;14(8):e0219899. doi: 10.1371/journal.pone.0219899 (PMC6687136; doi:10.1371/journal.pone.0219899)
Supplement: S2 Table — (DOCX) [file pone.0219899.s002.docx]

S2 Table. Relative risk ratio (95% confidence interval) of mild hypo- (<4.0 mmol/L) and hyperkalemia (≥5.0 mmol/L) for potential correlates (N=142,087).

| **Potential correlates** | **K<4.0** | **Z** | **K≥5.0** | **Z** |
| --- | --- | --- | --- | --- |
| Age (per 10 year increment) | 0.90 (0.89,0.91) | -14.61 | 0.91 (0.89,0.94) | -7.39 |
| Female | 1.49 (1.38,1.62) | 9.91 | 0.75 (0.63,0.90) | -3.06 |
| Black race | 1.72 (1.67,1.78) | 32.35 | 0.64 (0.60,0.69) | -11.78 |
| eGFR<60 (per 15 mL/min/1.73 m^2^ decrement) | 0.88 (0.85,0.90) | -8.98 | 1.68 (1.62,1.73) | 31.59 |
| eGFR≥60 (per 15 mL/min/1.73 m^2^ decrement) | 0.89 (0.88,0.90) | -15.16 | 1.34 (1.30,1.38) | 18.72 |
| Systolic blood pressure (per 20 mmHg increment) | 1.15 (1.13,1.16) | 21.28 | 0.98 (0.96,1.00) | -1.69 |
| Body mass index (per 5 kg/m^2^ increment | 0.99 (0.98,1.00) | -2.66 | 0.91 (0.89,0.93) | -10.54 |
| Diabetes | 0.99 (0.95,1.03) | -0.61 | 1.22 (1.14,1.30) | 6.10 |
| History of coronary artery disease | 0.89 (0.87,0.92) | -8.16 | 0.96 (0.91,1.01) | -1.69 |
| History of cerebrovascular disease | 1.04 (1.01,1.07) | 2.43 | 0.92 (0.87,0.97) | -3.20 |
| History of peripheral artery disease | 0.88 (0.85,0.90) | -8.06 | 1.09 (1.04,1.15) | 3.48 |
| History of atrial fibrillation | 1.10 (1.07,1.14) | 6.51 | 0.89 (0.84,0.93) | -4.55 |
| Use of ACEI/ARB | 0.74 (0.72,0.76) | -20.62 | 1.18 (1.12,1.24) | 6.40 |
| Use of loop/thiazide diuretics | 1.65 (1.60,1.70) | 34.98 | 0.75 (0.72,0.79) | -12.01 |
| Use of K-sparing diuretics | 1.24 (1.19,1.30) | 10.13 | 1.20 (1.12,1.29) | 5.14 |
| Use of beta-blockers | 0.91 (0.88,0.93) | -6.69 | 1.01 (0.97,1.06) | 0.56 |
| Use of other anti-hypertensive medications | 1.44 (1.40,1.48) | 27.04 | 0.82 (0.79,0.86) | -8.40 |
| Use of insulin | 0.86 (0.83,0.89) | -8.29 | 1.14 (1.07,1.20) | 4.47 |
| Use of other anti-diabetic medications | 0.82 (0.79,0.85) | -10.37 | 1.20 (1.13,1.27) | 6.03 |
| Use of statins | 0.98 (0.95,1.01) | -1.56 | 0.95 (0.91,0.99) | -2.43 |
| Use of anti-arrhythmic drugs | 0.96 (0.90,1.03) | -1.12 | 0.87 (0.78,0.97) | -2.52 |
| Use of digoxin | 0.82 (0.78,0.85) | -8.91 | 1.09 (1.02,1.17) | 2.58 |
| eGFR=estimated glomerular filtration rate, ACEI=angiotensin-converting enzyme inhibitor, ARB=angiotensin receptor blockers, K=potassium. | | | | |
