# Supplementary material for: Dyskalemia, its patterns, and prognosis among patients with incident heart failure: A nationwide study of US veterans
Source: PLoS One. 2019 Aug 8;14(8):e0219899. doi: 10.1371/journal.pone.0219899 (PMC6687136; doi:10.1371/journal.pone.0219899)
Supplement: S3 Table — (DOCX) [file pone.0219899.s003.docx]

S3 Table. Hazard ratio (95% confidence interval) of mortality for transient, intermittent, and persistent mild hypo- and hyperkalemia over one year after incident heart failure (no hypo- or hyperkalemia as referent) (N=118,477).

| **Model** | **K level (mmol/L)** | **Dyskalemia pattern** | | | |
| --- | --- | --- | --- | --- | --- |
|  |  | **0 (no)** | **1 (transient)** | **2 (intermittent)** | **3 (persistent)** |
| 1 | K<4.0 | Referent | 1.08 (1.05,1.11) | 1.22 (1.17,1.26) | 1.05 (1.02,1.08) |
| 2 | K<4.0 | Referent | 1.12 (1.09,1.16) | 1.28 (1.24,1.33) | 1.18 (1.15,1.21) |
| 1 | K≥5.0 | Referent | 1.25 (1.21,1.29) | 1.39 (1.32,1.45) | 1.39 (1.32,1.46) |
| 2 | K≥5.0 | Referent | 1.18 (1.14,1.21) | 1.26 (1.20,1.32) | 1.23 (1.18,1.30) |
|  |  |  |  |  |  |
| Model 1 adjusted for frequency of potassium measurement. | | | | | |
| Model 2 additionally adjusted for age, gender, race, estimated glomerular filtration rate, systolic blood pressure, body mass index, diabetes, history of coronary artery disease, history of cerebrovascular disease, history of peripheral artery disease, and history of atrial fibrillation, use of ACEI/ARB, use of loop/thiazide diuretics, use of K-sparing diuretics, use of beta-blockers, use of other anti-hypertensive medications, use of insulin, use of other anti-diabetic medications, use of statins, use of anti-arrhythmic drugs, and use of digoxin. | | | | | |
| ACEI=angiotensin-converting enzyme inhibitor, ARB=angiotensin receptor blockers, K=potassium. | | | | | |
