# Supplementary material for: Dyskalemia, its patterns, and prognosis among patients with incident heart failure: A nationwide study of US veterans
Source: PLoS One. 2019 Aug 8;14(8):e0219899. doi: 10.1371/journal.pone.0219899 (PMC6687136; doi:10.1371/journal.pone.0219899)
Supplement: S5 Table — (DOCX) [file pone.0219899.s005.docx]

S5 Table. Actions taken after experiencing hypo- and hyperkalemia over two years after incident heart failure.

|  | Hypokalemia (<3.5 mmol/L) | | Hyperkalemia (≥5.5 mmol/L) | |
| --- | --- | --- | --- | --- |
| Action taken | Case | Control | Case | Control |
| Emergency room visit within 7 days | 14.8% (1230/8296) ^‡^ | 4.8% (2497/51621) | 14.0% (903/6457) ^‡^ | 6.4% (5681/88739) |
| Repeated potassium measurement within 14 days | 27.5% (2285/8296) ^‡^ | 7.4% (3823/51621) | 42.5% (2743/6457) ^‡^ | 8.5% (7517/88739) |
| Discontinuation of ACEI/ARB within 60 days | 12.8% (525/4098) ^‡^ | 8.2% (2374/28928) | 13.2% (552/4173) ^‡^ | 7.9% (3747/47604) |
| Initiation of ACEI/ARB within 60 days | 10.8% (452/4198) | 10.4% (2354/22693) | 9.7% (222/2284) | 10.4% (4279/41135) |
| Discontinuation of diuretics within 60 days | 14.5% (754/5215) ^‡^ | 11.9% (2680/22486) | 15.5% (550/3557) ^‡^ | 11.9% (5161/43480) |
| Initiation of diuretics within 60 days | 15.2% (467/3081) ^‡^ | 10.1% (2955/29135) | 14.5% (420/2900) ^‡^ | 11.2% (5080/45259) |
| Discontinuation of K-sparing diuretics within 60 days | 20.0% (156/780) ^‡^ | 13.7% (643/4708) | 25.6 % (332/1298) ^‡^ | 12.8% (1017/7940) |
| Initiation of K-sparing diuretics within 60 days | 4.8% (364/7516) ^‡^ | 1.6% (742/46913) | 2.2% (116/5159) | 2.1% (1731/80799) |
| Discontinuation of beta-blockers within 60 days | 8.7% (425/4877) ^‡^ | 6.8% (1984/29079) | 7.1% (294/4169) | 7.0% (3420/48836) |
| Initiation of beta-blockers within 60 days | 11.3% (385/3419) | 10.5% (2358/22542) | 11.4% (261/2288) | 10.3% (4110/39903) |
| Initiation of kayexalate within 60 days | 1.0% (11/1115) ^‡^ | 3.0% (173/5701) | 19.9% (403/2026) ^‡^ | 0.1% (4/5655) |
| Initiation of K supplement within 60 days | 14.2% (605/4256) ^‡^ | 4.2% (663/15635) | 3.1% (77/2455) ^†^ | 4.3% (1547/35596) |
|  |  |  |  |  |
| ACEI=angiotensin-converting enzyme inhibitor, ARB=angiotensin receptor blockers, K=potassium. | | | | |
| ^*^ p<0.05, ^†^p<0.01, ^‡^p<0.001 | | | | |
