# Supplementary figures and images for: Dyskalemia, its patterns, and prognosis among patients with incident heart failure: A nationwide study of US veterans
Source: PLoS One. 2019 Aug 8;14(8):e0219899. doi: 10.1371/journal.pone.0219899 (PMC6687136; doi:10.1371/journal.pone.0219899)

S1 Fig. Study flow diagram
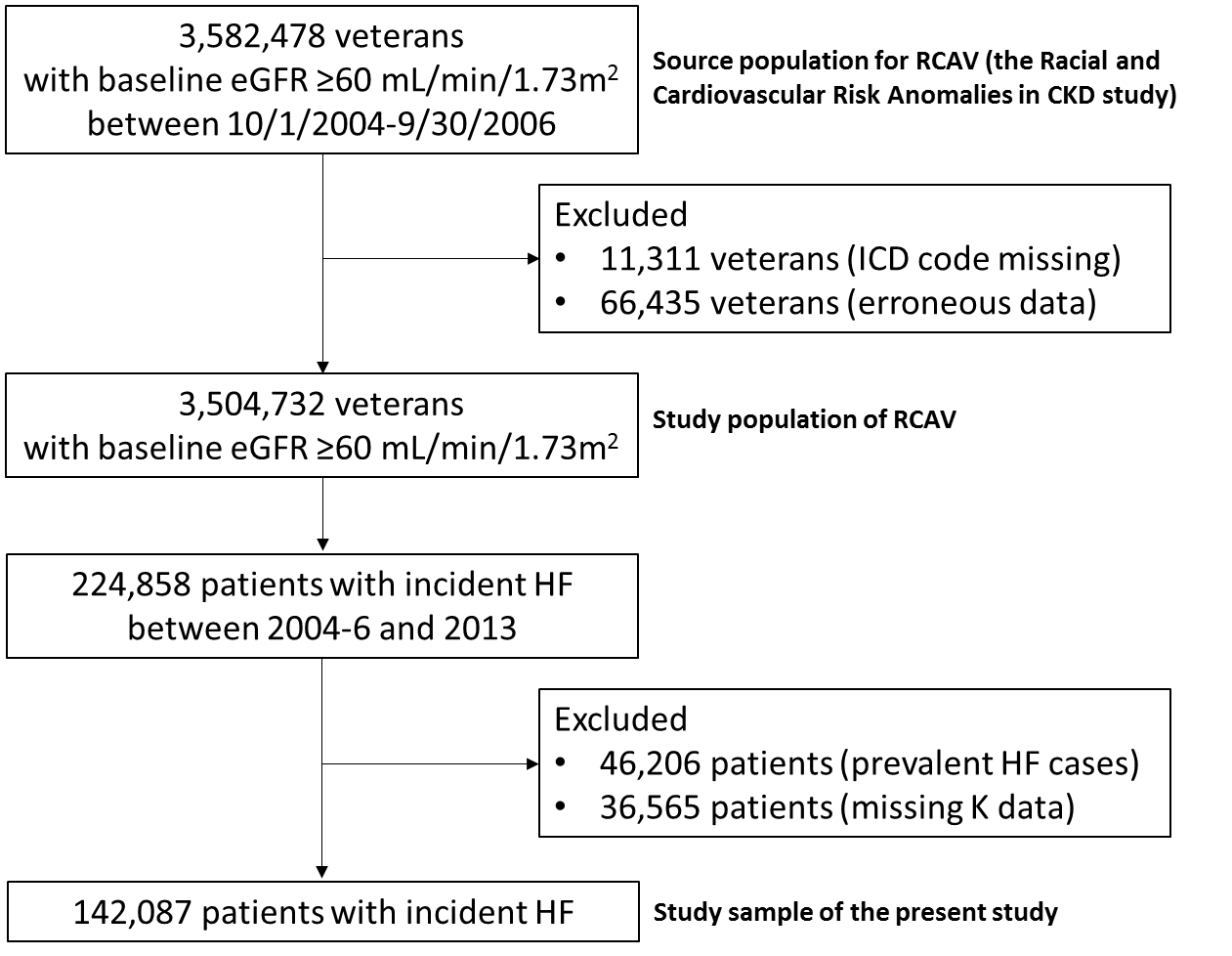

Supplement: S1 Fig — (DOCX) [file pone.0219899.s006.docx]

S2 Fig. Kaplan-Meier survival estimates after incident HF


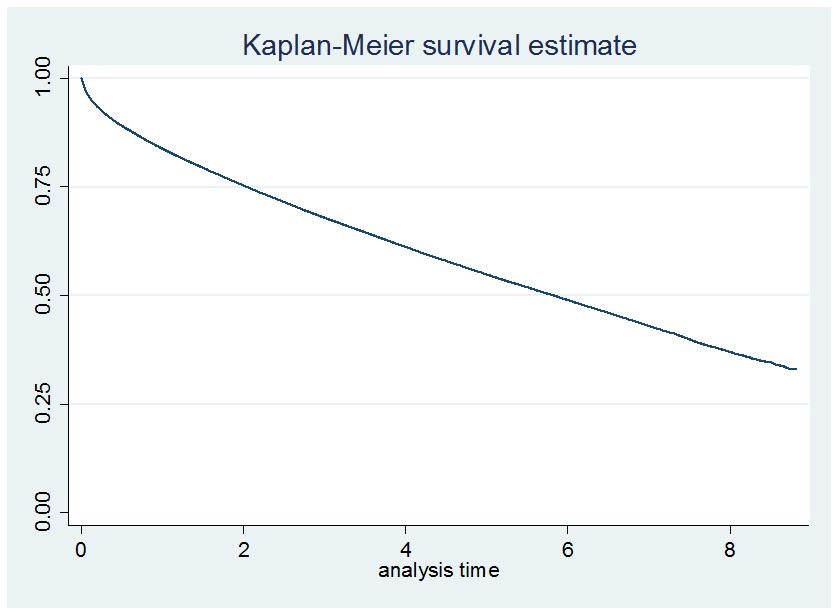

Supplement: S2 Fig — (DOCX) [file pone.0219899.s007.docx]
