# Supplementary material for: Dyskalemia, its patterns, and prognosis among patients with incident heart failure: A nationwide study of US veterans
Source: PLoS One. 2019 Aug 8;14(8):e0219899. doi: 10.1371/journal.pone.0219899 (PMC6687136; doi:10.1371/journal.pone.0219899)
Supplement: S4 Fig — (DOCX) [file pone.0219899.s009.docx]

S4 Fig. Unadjusted (left) and adjusted* (right) hazard ratio of mortality after incident heart failure according to baseline serum potassium levels in the range of 0.02 to 99.8 percentiles by the use of potassium-sparing diuretics. Diamond indicates the reference point at 4.2 mmol/L; dot, statistical significance compared to the reference point; shade, 95% confidence intervals. Knots were put at 3.6, 4.0, 4.2, 4.4, 4.6, and 5.0 mmol/L. *Adjusted for age, gender, race, blood pressure, body mass index, diabetes, a history of coronary heart disease, stroke, peripheral artery disease, and atrial fibrillation, and use of angiotensin-converting enzyme inhibitor/angiotensin receptor blockers, potassium-wasting diuretics (loop and thiazide), potassium-sparing diuretics, beta-blockers, use of other anti-hypertensive medications, insulin, other anti-diabetic medications, statins, digoxin, and anti-arrhythmic medication.

No use of potassium sparing diuretics

Use of potassium sparing diuretics
